# DNA Barcoding of Portuguese Lacewings (Neuroptera) and Snakeflies (Raphidioptera) (Insecta, Neuropterida)

**DOI:** 10.3897/zookeys.1054.64608

**Published:** 2021-08-03

**Authors:** Daniel Oliveira, Cátia Chaves, Joana Pinto, Joana Paupério, Nuno Fonseca, Pedro Beja, Sónia Ferreira

**Affiliations:** 1 CIBIO/InBIO – Centro de Investigação em Biodiversidade e Recursos Genéticos da Universidade do Porto, Vairão, 4485–661 Vairão, Portugal Centro de Investigação em Biodiversidade e Recursos Genéticos da Universidade do Porto Vairão Portugal; 2 Departamento de Biologia, Faculdade de Ciências, Universidade do Porto, 4169-007 Porto, Portugal Universidade do Porto Porto Portugal; 3 CIBIO/InBIO, Centro de Investigação em Biodiversidade e Recursos Genéticos, Instituto Superior de Agronomia, Universidade de Lisboa, Lisboa, Portugal Universidade de Lisboa Lisboa Portugal

**Keywords:** Cytochrome c oxidase subunit I (COI), DNA barcode, mitochondrial DNA, Portugal, taxonomy

## Abstract

The orders Neuroptera and Raphidioptera include the species of insects known as lacewings and snakeflies, respectively. In Portugal, these groups account for over 100 species, some of which are very difficult to identify by morphological analysis. This work is the first to sample and DNA sequence lacewings and snakeflies of Portugal. A reference collection was built with captured specimens that were identified morphologically. DNA barcode sequences of 658 bp were obtained from 243 specimens of 54 species. The results showed that most species can be successfully identified through DNA barcoding, with the exception of seven species of Chrysopidae (Neuroptera). Additionally, the first published distribution data are presented for Portugal for the neuropterans *Gymnocnemiavariegata* (Schneider, 1845) and Myrmecaelurus (Myrmecaelurus) trigrammus (Pallas, 1771).

## Introduction

Neuropterida is a superorder of insects which encompasses the orders Neuroptera, Raphidioptera and Megaloptera. The present work focuses on DNA barcoding of the first two orders in Portugal, while DNA barcoding of Megaloptera in the country was addressed in Ferreira et al. (2019).

The order Neuroptera includes the holometabolous insects commonly known as lacewings. With at least 6000 species worldwide, more than 300 of which occur in Europe, Neuroptera accounts for most of the diversity of the Neuropterida (Aspöck 2002b; Aspöck et al. 2015). For the almost 200 species known in the Iberian Peninsula, around half have been recorded in Portugal, spanning 10 families (Aspöck et al. 2001; Letardi and Almeida 2013; Monserrat and Triviño 2013; Oliveira and Ferreira 2020; this work).

The small order Raphidioptera Latreille, 1810, groups about 260 species of insects worldwide (Aspöck 2002a), which are commonly known as snakeflies. From the 16 species of Raphidioptera present in the Iberian Peninsula, six species are known to occur in Portugal (Monserrat and Papenberg 2015; Papenberg 2015). The family Raphidiidae is represented by five species: *Atlantoraphidiamaculicollis* (Stephens, 1836), *Harraphidialaufferi* (Navás, 1915), *Hispanoraphidiacastellana* (Navás, 1915), *Ohmellabolivari* (Navás, 1915) and *Subillaaliena* (Navás, 1915). In contrast, Inocelliidae is represented by a single species: *Fiblahesperica* Navás, 1915 (Monserrat and Papenberg 2015; Papenberg 2015).

The monophyly of the three orders of Neuropterida (Megaloptera as a sister group of Neuroptera + Raphidioptera) has been solidly established. Nonetheless, taxonomy of the groups is incompletely resolved and internal relationships are not yet established, despite recent studies, especially in the case of Neuroptera (Aspöck 2002b; Winterton et al. 2010; Wang et al. 2017; Engel et al. 2018). Most notably, recent evidence has been mounting for the integration of Ascalaphidae as a subfamily of Myrmeleontidae (Winterton et al. 2018; Machado et al. 2019; Vasilikopoulos et al. 2020).

DNA barcoding was proposed in 2003 as a method to rapidly and accurately identify species (Hebert et al. 2003; Hebert and Gregory 2005). This method relies on the existence of comprehensive databases of short DNA sequences (the DNA barcodes), which are attributed to previously identified specimens and used for comparison with DNA barcode sequences obtained from unidentified specimens or even environmental samples. For insects, the typical DNA barcode consists of a 658 bp sequence of the cytochrome c oxidase subunit I (COI) (Folmer et al. 1994), also known as the “Folmer region”. DNA barcoding has been used in studies involving Neuropterida, namely in the construction and analysis of DNA barcode databases for the fauna of certain regions, including Central Europe (Morinière et al. 2014) and Beijing, China (Yi et al. 2018), in the description of new species (Pantaleoni and Badano 2012; Badano et al. 2016), and to resolve taxonomic questions (Price et al. 2015). It is important to accurately identify species, especially the ones with agricultural applications, such as those in Chrysopidae and Hemerobiidae, as misidentifications may compromise biological control. Hitherto, DNA barcoding studies of Neuroptera and Raphidioptera in Portugal were non-existent, despite the considerable number of species known to occur in the country.

In this work, we present a contribution to the DNA barcode library for the Portuguese species of Neuroptera and Raphidioptera representing about 50% of known species in the country, alongside new and interesting distributional data. While most species were found to be identifiable through the use of the obtained DNA barcodes, this was not true for some cases in Chrysopidae. This work was conducted within the frame of the InBIO Barcoding Initiative, which aims at producing a comprehensive DNA barcode database for the Portuguese terrestrial invertebrate biodiversity.

## Materials and methods

### Sampling of specimens

Specimens were collected during field expeditions throughout continental Portugal, from 2006 to 2019, and stored in 96% ethanol at the InBIO Barcoding Initiative reference collection (Vairão, Portugal). Specimens were captured during direct searches of the environment or lured by light trapping, the latter with UV LEDs or mercury vapour lamps. Morphological identification was done based on the most recent literature on Iberian Neuroptera and Raphidioptera (Monserrat and Acevedo 2012a, b, 2013; Monserrat 2014a, b, c, 2016a, b; Monserrat et al. 2014; Monserrat and Papenberg 2015), and using an Olympus SZX2-ILLT Stereozoom microscope when necessary. From each specimen, one tissue sample (a leg) was removed and stored in 96% ethanol for DNA extraction.

### DNA extraction, amplification and sequencing

For each species, we selected six specimens for DNA sequencing based on their location of capture, attempting to maximize the geographical coverage of the study. For species with less than six specimens, all were selected for sequencing.

DNA was extracted from most tissue samples using the EasySpin Genomic DNA Microplate Tissue Kit. For specimens belonging to species of smaller sizes (such as those from the Hemerobiidae and Coniopterygidae families), the QIAmp DNA Micro Kit was used, as it is designed to extract higher concentrations of genetic material from samples with small amounts of DNA.

Amplification of the DNA was performed using three different primer pairs, that amplify three overlapping fragments of the same 658 bp region of the COI mitochondrial gene. Initially, we used two primer pairs, LCO1490 (Folmer et al. 1994) + Ill_C_R (Shokralla et al. 2015) and Ill_B_F (Shokralla et al. 2015) + HCO2198 (Folmer et al. 1994) (henceforth referred to as LC and BH, respectively) to amplify two overlapping fragments of 325 bp and 418 bp, respectively. Following publication of the third primer pair, BF2 + BR2 (422 bp fragment), by Elbrecht and Leese (2017), this started to be used instead of Ill_B_F + HCO2198 due to higher amplification efficiency.

PCRs were performed in 10 µl reactions, containing 5 µl of Multiplex PCR Master Mix (Qiagen, Hilde, Germany, 0.3 (BF2-BR2) – 0.4 mM of each primer, and 1–2 µl of DNA, with the remaining volume in water. For DNA amplification, an initial denaturation at 95 °C for 15 min was performed followed by 5 cycles at 95 °C for 30 sec, 47 °C for 45 sec, 72 °C for 45 sec (only for LC and BH); then 40 cycles at 95 °C for 30 sec, 51 °C for 45 sec (48 °C for 60 sec for BF2 + BR2), 72 °C for 45 sec; and a final elongation step at 60 °C for 10 min. DNA amplification was performed in T100 Thermal Cycler (Bio-Rad, California, USA).

All PCR products were analysed by agarose gel electrophoresis and samples selected for sequencing were then organised for assignment of sequencing ‘indexes’. One of two types of index were used for each run. For Illumina indexes, samples were pooled into one plate, as described in Shokralla et al. (2015). When using custom indexes (designed based on (Meyer and Kircher (2010)) no pooling was required. The latter allow for a maximum of 1920 unique index combinations. A second PCR was then performed where the ‘indexes’ and Illumina sequencing adapters were attached to the DNA extract. The index PCR was performed in a volume of 10 µl, including 5 µL of Phusion High-Fidelity PCR Kit (New England Biolabs) or KAPA HiFi PCR Kit (KAPA Biosystems, USA), 0.5 µL of each ‘index’ and 2 µL of diluted PCR product (usually 1:4). This PCR reaction is only of 10 cycles and performed at an annealing temperature of 55 °C. The amplicons were purified using AMPure XP beads (New England Biolabs) before quantification using NanoDrop 1000 (Thermo Scientific). This step allows for a normalization of concentrations between samples before the final quantification step with a qPCR using the KAPA Library Quantification Kit Illumina Platforms (KAPA Biosystems, USA) (Paupério et al. 2018).

Sequencing was performed at the CIBIO facilities on an Illumina MiSeq benchtop system, using a V2 MiSeq sequencing kit (2× 250 bp).

### Bioinformatic processing and data analysis

Sequences were filtered and processed with OBITools (Boyer et al. 2014) and the fragments were assembled into their consensus 658 bp-long sequences using Geneious 9.1.8 (https://www.geneious.com). The obtained DNA sequences were then compared against the BOLD database (Ratnasingham and Hebert 2007) using the built-in identification engine, based on the BLAST algorithm. Sequences were submitted to the BOLD database and the Barcode Index Numbers (BIN) for every sequence were retrieved and analysed (Suppl. material 1: Table S1).

All DNA barcode sequences were aligned in Geneious 9.1.8. with the CLUSTALW (Thompson et al. 1994) plugin. Nucleotide composition of all sequences, as well as intra and interspecific p-distances were calculated in MEGAX (Kumar et al. 2018). Neighbour-joining trees were constructed in PAUP* 4.0a167 (Swofford 2003), with 1000 bootstrap replicates, as a simple way of visualizing genetic distance between sequences, while detecting possible misidentifications and incongruences. First, a tree with all obtained DNA barcode sequences of Neuroptera and Raphidioptera was constructed. For this, the outgroup sequences IBIMP001-19 and AGRID020-10 from the BOLD database (of *Sialisfuliginosa* Latreille, 1803 and *Agriotesproximus* Schwarz, 1891, respectively) were used to root the tree. These outgroups refer, respectively, to a species of Megaloptera (the third order within the Neuropterida) and a species of Coleoptera, the closest order to Neuropterida (Wang et al. 2017). Additionally, a NJ tree was constructed for Chrysopidae and Hemerobiidae, utilizing the sequences FBNE073-11 and FBNE001-11 (of *Osmylusfulvicephalus* (Scopoli, 1763) and *Sisyranigra* (Retzius, 1783), respectively) as outgroups. The latter set of outgroups was used for family-level trees as representative of Osmylidae Linnaeus, 1758 and Sisyridae Banks, 1905.

An analysis of the data with the Automatic Barcode Gap Discovery (ABGD) method (Puillandre et al. 2012) was performed at the dedicated website (https://bioinfo.mnhn.fr/abi/public/abgd/abgdweb.html), as a test of the existence of a barcoding gap between species, which is fundamental to species identification using DNA barcodes (Hebert et al. 2003, 2004).

## Results

**Figure 1.**
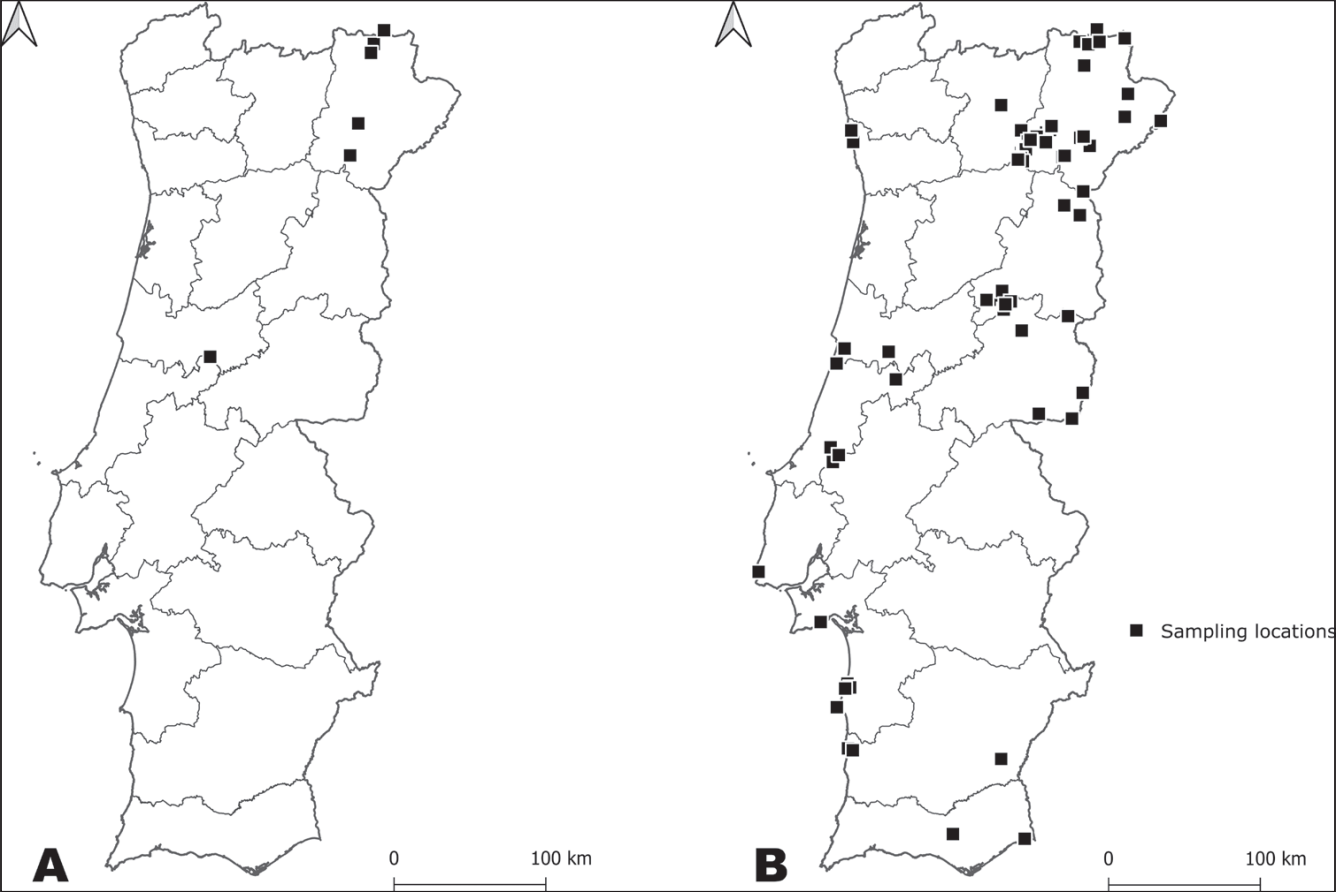
Map of continental Portugal with sampling locations **A** sampling locations of the 8 captured specimens of Raphidioptera (*N* = 8) **B** sampling locations of the 235 captured specimens of Neuroptera (*N* = 67).

DNA barcode sequences of 658 bp were obtained for 243 specimens of Neuropterida, representing 54 of the 104 species known to occur in continental Portugal (Fig. 1; Suppl. material 1: Table S1). These species are representative of 9 of 10 families of Neuroptera, and one of two families of Raphidioptera recorded in the country. These sequences represent 21 new species of Neuroptera and one of Raphidioptera for the BOLD database. Furthermore, of the already available sequences in BOLD only six originate from continental Portugal (accessed on 19/01/2021).

### Neuroptera Linnaeus, 1758

Neuroptera specimens were collected from 67 sampling locations, in 12 districts (Fig. 1 and Suppl. material 1: Table S1). From the 51 species, 12 were captured only once and are therefore represented by a single DNA barcode sequence in the dataset. Two of the species were hitherto without published records in scientific literature for the country: *Gymnocnemiavariegata* and *Myrmecaelurustrigrammus* (Suppl. material 1: Table S1), despite being widespread in the whole Euro-Mediterranean area and their presence well known in Spain.

For the DNA barcode sequences of Neuroptera, average nucleotide composition is 39.4% thymine (T), 16.2% cytosine (C), 28.6% adenine (A) and 15.8% guanine (G). Base frequencies analysis revealed GC-contents of 32% for the DNA barcode fragment. Average genetic p-distances between captured species ranged from 0.46% between *Pseudomalladapicteti* (McLachlan, 1880) and *Pseudomalladaflavifrons* (Brauer, 1851) to 25.91% between *Dilarmeridionalis* Hagen, 1866 and *Aleuropteryxiberica* Monserrat, 1977 (Suppl. material 2: Table S2). Intraspecific distances ranged from 0% in *Palpareshispanus* Hagen, 1860 (*N* = 3), *Cunctochrysabaetica* (Hölzel, 1972) (*N* = 5) and *Italochrysaitalica* (Rossi, 1790) (*N* = 2) to 3.6% in *Dilarmeridionalis* (*N* = 4) (Suppl. material 2: Table S2).

**Figure 2.**
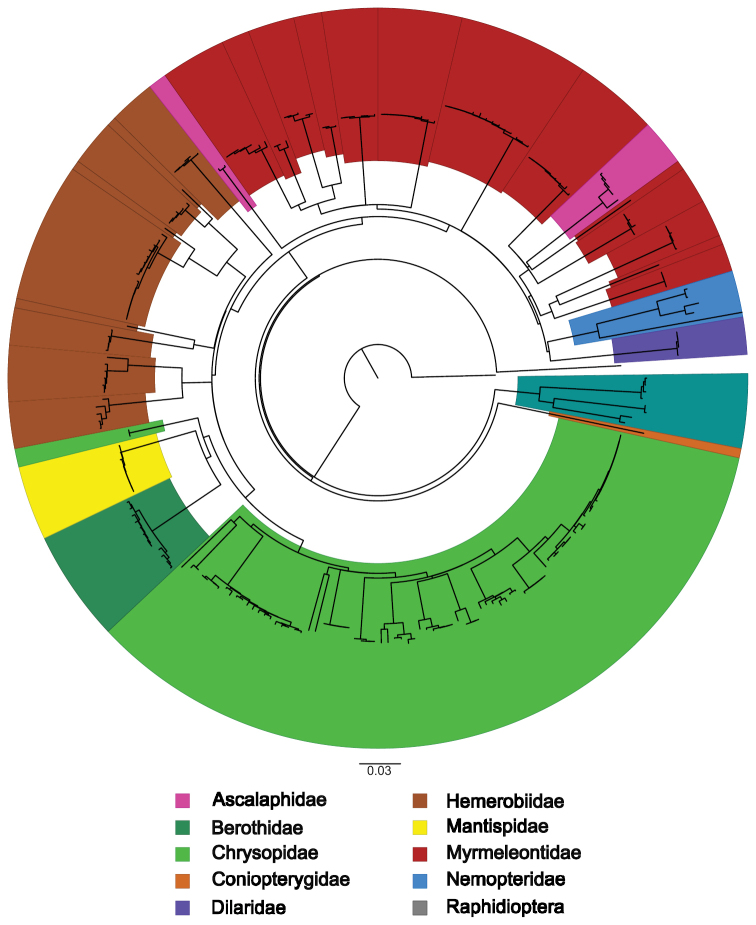
Neighbour-joining tree of all obtained DNA sequences for Portuguese Neuroptera and Raphidioptera. Neighbour-joining tree constructed in PAUP* 4.0a167. Non-highlighted terminal branches represent the two outgroup sequences.

**Figure 3.**
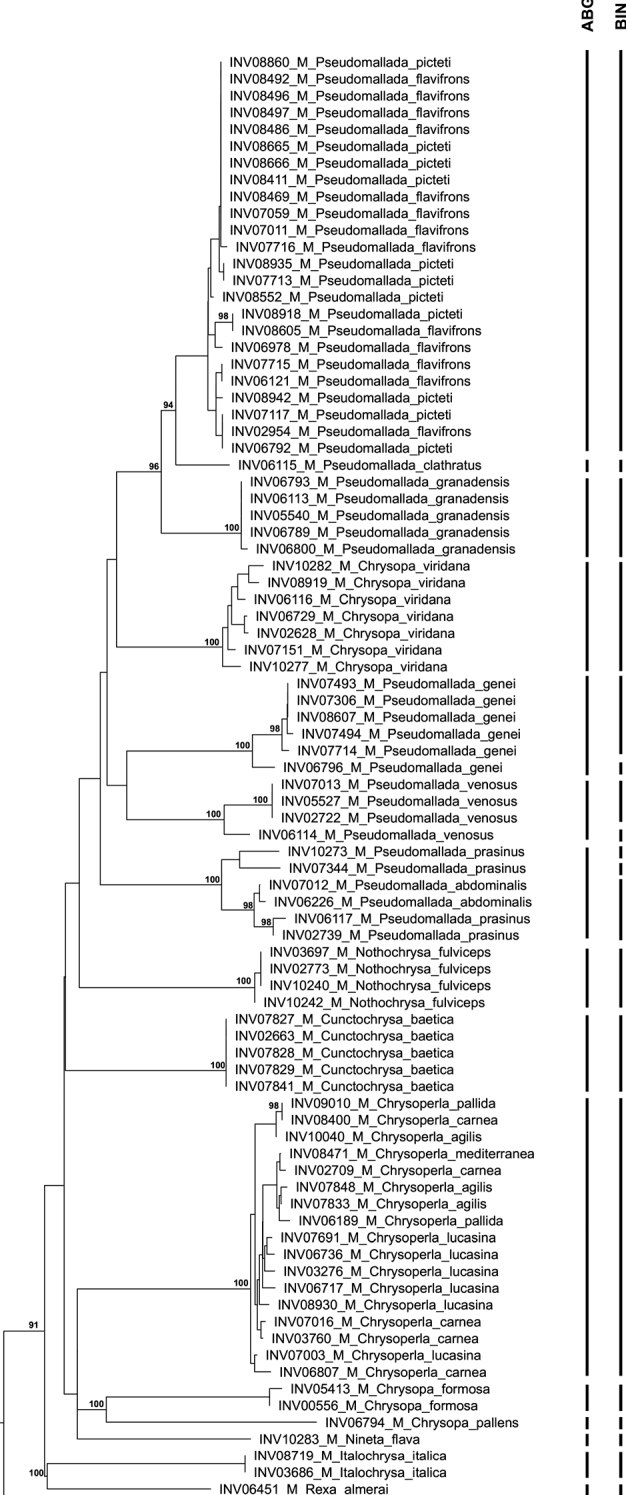
Neighbour-joining tree of Chrysopidae DNA barcode sequences. Neighbour-joining tree constructed in PAUP* 4.0a167 and contrasted with the results from the ABGD analysis and BIN attribution. Bootstrap values under 90% omitted.

Regarding the neighbour-joining analysis (Fig. 2), most species were recovered as monophyletic except for seven species of Chrysopidae, which were separated into two polyphyletic groups of morphologically identified species. One group encompassing *P.picteti* and *P.flavifrons*, another encompassing *Chrysoperlacarnea* (Stephens, 1836), *Chrysoperlalucasina* (Lacroix, 1912), *Chrysoperlapallida*Henry et al., 2002, *Chrysoperlaagilis*Henry et al., 2003 and *Chrysoperlamediterranea* (Hölzel, 1972) (Fig. 3).

**Figure 4.**
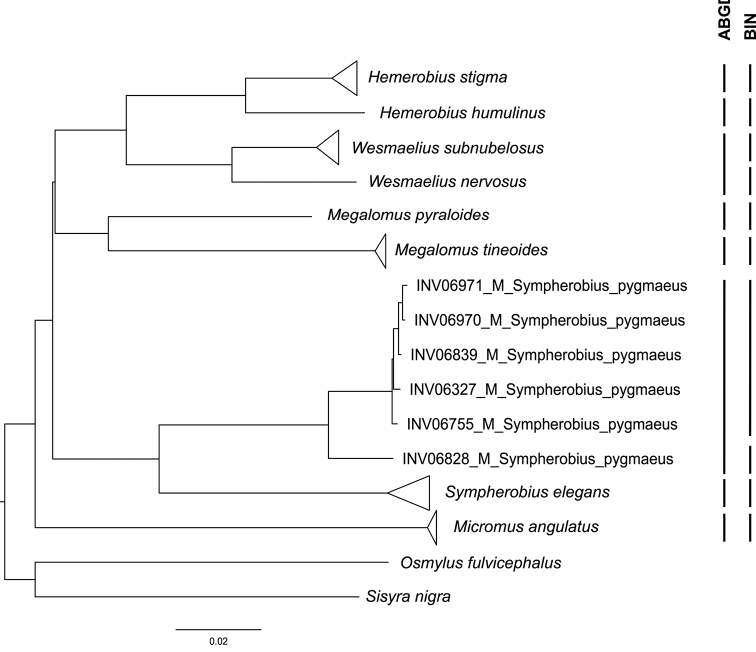
Neighbour-joining tree of Hemerobiidae DNA barcode sequences. Neighbour-joining tree constructed in PAUP* 4.0a167 and contrasted with the results from the ABGD analysis and BIN attribution. Bootstrap values under 90% omitted. Subtrees were collapsed for the monophyletic morphologically identified species. Triangle size for each species is proportional to the intraspecific distance.

The ABGD method yielded partitions generally congruent with morphological identification. Nonetheless, some exceptions were noted. Regarding the Chrysopidae, the ABGD analysis yielded 15 partitions (P = 0.0055) (Fig. 3). While congruent with the NJ analysis (by considering the aforementioned polyphyletic groups of species as two separate species), it also grouped the DNA barcoding sequences of *Pseudomalladaprasinus* and *Pseudomalladaabdominalis* (Brauer, 1856), which NJ analysis separates into three clades (in congruence with three detected morphospecies; see Discussion), into one single “species”. In the Hemerobiidae family, the ABGD analysis recovered only eight partitions (P = 0.0492), grouping *Wesmaeliussubnebulosus* (Stephens, 1836) and *Wesmaeliusnervosus* (Fabricius, 1793) (Fig. 4).

Similar to the other two methods used, BIN allocation using BOLD Systems yielded congruent results for most species, with some particular cases of incongruence. In Ascalaphidae, the sequences belonging to the two species of *Libelloides* were grouped under the same BIN. For Chrysopidae, the BIN framework clustered sequences similarly to ABGD, except for two sequences of *Pseudomalladaprasinus* (INV10273 and INV07344), which were assigned BINs different from each other and the other sequences for the species, as well as one sequence from both *Pseudomalladagenei* and *Pseudomalladavenosus* which were not grouped in the same BIN as the other sequences of the same species (Fig. 3 and Suppl. material 1: Table S1). The sequences of Hemerobiidae yielded 10 BINs, one more than the number of morphologically identified species, as sequences of *Sympherobiuspygmaeus* are in two BINs (Fig. 4).

### Raphidioptera Latreille, 1810

DNA barcode sequences were obtained for eight specimens of Raphidioptera, accounting for three of the six species known to occur in Portugal.

Average nucleotide composition of all DNA barcode sequences of Raphidioptera was calculated as 37.2% thymine (T), 18.1% cytosine (C), 29.6% adenine (A) and 15.1% guanine (G). Base frequencies analysis revealed GC-contents of 33% for the DNA barcode fragment. Genetic distances between species ranged from 12.3% between *A.maculicollis* and *H.castellana* to 15.9% between *H.laufferi* and *H.castellana.* Intraspecific distances ranged from 0.2% in *H.castellana* to 1.2% in *A.maculicollis* (Suppl. material 2: Table S2). The NJ tree constructed with the calculated genetic distances recovered all species as monophyletic (Fig. 2). Analysis with the BOLD BIN system yielded three BINs, congruent with the morphological identification. Similarly, three partitions were recovered from ABGD analysis.

The eight specimens of Raphidioptera were captured in six sampling locations in Bragança and Leiria (Fig. 1 and Suppl. material 1: Table S1)

## Discussion

In this work, DNA barcode sequences and their respective analyses, as well as novel distributional data are provided based on 235 specimens of 51 species of Neuroptera and 8 specimens of 3 species of Raphidioptera. This is the first study focusing on DNA barcoding for these orders in Portugal.

The main goal of this work was to compile a DNA barcode reference collection for the Portuguese species of Neuroptera and Raphidioptera. About 50% of the faunal diversity of the groups is represented in the collection, and DNA barcode sequences were added to the BOLD database for species hitherto unrepresented. The analyses conducted suggest that most of the encompassed species can be identified with the COI gene-based DNA barcodes. This is the case for the Ascalaphidae, Berothidae, Mantispidae, Myrmeleontidae and Nemopteridae families. For the other families, Chrysopidae and Hemerobiidae, further scrutiny is necessary.

Interestingly, despite the congruence of taxonomy and the obtained DNA barcodes for the families Ascalaphidae and Myrmeleontidae, the genetic distances and phylogenetic tree (Fig. 2) show the latter group as paraphyletic. These results may provide further evidence for the integration of current Ascalaphidae species into the family Myrmeleontidae, a taxonomic change that has seen growing support in recent years (Winterton et al. 2018; Machado et al. 2019; Vasilikopoulos et al. 2020)

Regarding the Chrysopidae, the results show four groups of species with conflicting results between morphological identification, NJ and ABGD analysis, and BIN attribution. The first consists of the DNA barcode sequences belonging to *P.flavifrons* and *P.picteti*, whose sequences were recovered as a single clade (NJ) and placed by ABGD analysis into a single group. Despite possessing distinctive morphological characteristics these are closely-related species with high degree of morphological variation (Aspöck et al. 2001; Monserrat 2016b; Duelli et al. 2017). The obtained results suggest that *P.picteti* and *P.flavifrons* share mitochondrial haplotypes, which may be due to incomplete lineage sorting or mitochondrial genome capture as a result of introgressive hybridization.

The morphospecies *P.venosus* and *P.genei* were recovered as monophyletic and ABGD considered each of the species as single units, although two different BINs were attributed to each species.

The *Pseudomalla* “*prasinus*” species complex, where *P.prasinus* and *P.abdominalis* are included, is the third group with conflicting results between NJ, ABGD and morphological analysis, and has been a subject of interest and contention in Neuropterology for over a century (McLachlan 1886). Recent molecular genetics works have supported the existence of a species complex (Duelli et al. 2017), showing cryptic diversity in the group. One of the prasinoid morphotypes is known as “*marianus*” and was previously considered as a valid species. The specimens INV10273 and INV07344 were identified as *Pseudomalladamarianus*, by utilizing the key available for the Iberian Peninsula in Monserrat (2016b). Duelli and Obrist (2019) established the synonymy of *P.marianus* with *P.prasinus*, previously proposed by Hölzel (1973), based on Central European specimens. In the former, authors state that the morphological characters previously attributed to the “*marianus*” morph (i.e., larger size and bundled egg placement) are the ones that define the “real” *P.prasinus*. As such, smaller specimens belonging to the “*prasinus*” species complex can’t yet be identified conclusively to species level until the prasinoid morphotypes are well-defined and described as a species (Duelli and Obrist 2019). However, the implications of this work on the Iberian Peninsula’s specimens of the “*prasinus*” species complex are not clear and require further research. In the present work, the NJ analysis was congruent with the morphological identification based on the characteristics described in Monserrat (2016b) since it separately grouped INV7344 and INV10273, which were identified as the “*marianus*” morphotype, but failed to retrieve *P.prasinus* as monophyletic. In contrast, the ABGD analysis grouped all specimens of *P.prasinus* and *P.abdominalis*. Additionally, the intraspecific distance between DNA barcode sequences of *P.prasinus* (2.25%) was higher than expected relative to the other species in our dataset. Our results, albeit limited, provide additional support to the existence of cryptic diversity in *P.prasinus*. Identification through DNA barcoding may prove problematic until the taxonomy of the group is better resolved, and will likely benefit of the use of other DNA markers.

A more complex situation is that of *Chrysoperlacarnea*, *C.lucasina*, *C.pallida*, *C.agilis* and *C.mediterranea*, in which all obtained sequences are grouped by NJ, ABGD and BIN analysis in a single unit. The five species belong to the so-called *C.carnea* species complex (Thierry et al. 1998; Henry et al. 2002, 2013). So far, the most reliable way to identify the species in this group is by their substrate-borne vibrational songs, produced by tremulation (Henry and Wells 2015). Even though these are not used for attraction of mates at long-distances as in many other animals, these signals are produced for close-range recognition of sexually receptive mates (Henry et al. 2002, 2003, 2012). The obtained results for the species of the group are congruent with previous studies (Lourenço et al. 2006; Morinière et al. 2014) and might be a result of the pre-copulatory reproductive isolation and the recent and rapid speciation of this group of species (Henry et al. 2013). Considering the obtained data and the available literature, a COI-based DNA barcode is not a feasible tool for species identification in this species complex.

The analysis of the sequences obtained from Dilaridae specimens yielded the highest intra and interspecific genetic distances of all studied species. The intraspecific genetic diversity in *Dilarmeridionalis* was 3.67% (*N* = 4), while the genetic distance between the *D.meridionalis* and *D.saldubensis* was 17.7%. Since previous works on DNA barcoding of Neuroptera have poorly (Yi et al. 2018) or not represented (Morinière et al. 2014) the family at all, further sampling and sequencing would be needed to access the validity of DNA barcoding based on the COI gene for identification of species in this family.

The two species of *Wesmaelius* were separated in the NJ analysis as by morphology, though ABGD failed to recover two distinct groups. Furthermore, both the ID engine and BIN analysis in BOLD systems clearly separated the species and grouped the sequences in BINs with other sequences available in the BOLD database of the same two species. Considering these results, we suggest that COI DNA barcode sequences may be used in the identification of these two species.

Another species that presents more than one BIN is *Sympherobiuspygmaeus.* The genetic diversity observed is congruent with previous work (Morinière et al. 2014) and further research is needed to verify if it is a case of cryptic diversity.

In our dataset, all species of Raphidioptera showed relatively low intraspecific divergence when compared with the respective interspecific distances. Despite the low number of DNA barcode sequences available and the absence of three of the six species in the dataset, the obtained results suggest that a DNA barcoding approach using a COI gene fragment may be used to discern between species of Portuguese Raphidioptera. This assumption is reinforced by the fact that all six species in the country belong to six different genera and are, as such, predicted to show relatively high interspecific distances between them.

For the large majority of encompassed species, DNA barcoding appears to be a reliable method of identification. While DNA barcoding cannot replace morphological taxonomy experts entirely, especially in taxa where the taxonomy still needs revision, it can aid in species identification in cases where morphology cannot be used. For example, in diet analyses, where only small body parts (or none at all) can be retrieved, using DNA barcoding may be the only method suitable for species identification, allowing the understanding of species interactions and their roles in the ecosystems.

Currently 73 species of Neuropterida present in Portugal have DNA barcoding data available, comprising the 54 species encompassed in this work and the 19 already available in the BOLD database from other countries. Nonetheless, 29 species known to occur in Portugal remain without DNA barcode available and further efforts are needed to fill this gap.

## Conclusion

This work provides novel data on the DNA barcoding and geographical distribution of Neuroptera and Raphidioptera species in Portugal. Our results suggest that DNA barcoding using COI Folmer region may be used to identify the great majority of species of Neuroptera and Raphidioptera species recorded in the country. It is not, however, suitable for identification of several species of the Chrysopidae family. In total, there were 22 cases where the first publicly available DNA barcode sequence for a species was obtained but further sampling and sequencing efforts are still needed for many. The completion of DNA barcode databases is an ongoing effort and, in the cases of Neuroptera and Raphidioptera, still require much work, including in Europe, where several species are not yet sequenced. The future, however, looks bright as international initiatives are promoting and aiding in the development of DNA barcode sequences databases for particular regions worldwide (Letardi 2019).

## Supplementary materials

Supplementary material 1 Summary table of all used sequences and specimens, with country of origin Data type Occurences and access codes to DNA barcodes Explanation note For captured specimens, sex, latitude and longitude coordinates (WGS 84), date of capture and IBI reference collection code (IBI) are presented. BOLD accession numbers and BINs (when available) presented for all DNA sequences used.. https://binary.pensoft.net/file/573500This dataset is made available under the Open Database License (http://opendatacommons.org/licenses/odbl/1.0/). The Open Database License (ODbL) is a license agreement intended to allow users to freely share, modify, and use this Dataset while maintaining this same freedom for others, provided that the original source and author(s) are credited.Daniel Oliveira, Sónia Ferreira

Supplementary material 2 Genetic distances Data type Genetic distances between analysed specimens Explanation note Estimates of average genetic divergence (uncorrected p-distances) for species of Neuropterida. Values under the diagonal refer to interspecific divergence while values in the diagonal and in bold represent intraspecific divergence. https://binary.pensoft.net/file/573501This dataset is made available under the Open Database License (http://opendatacommons.org/licenses/odbl/1.0/). The Open Database License (ODbL) is a license agreement intended to allow users to freely share, modify, and use this Dataset while maintaining this same freedom for others, provided that the original source and author(s) are credited.Daniel Oliveira, Sónia Ferreira

## References

[B1] Aspöck H (2002a) The biology of Raphidioptera: A review of present knowledge. Acta Zoologica Academiae Scientiarum Hungaricae 48: 35–50.

[B2] Aspöck H Hölzel H Aspöck U (2001) Kommentierter Katalog der Neuropterida (Insecta: Raphidioptera, Megaloptera, Neuroptera) der Westpaläarktis. Denisia 2: 1–606.

[B3] Aspöck U (2002b) Phylogeny of the Neuropterida (Insecta: Holometabola). Zoologica Scripta 31: 51–55. 10.1046/j.0300-3256.2001.00087.x

[B4] Aspöck U Aspöck H Letardi A de Jong Y (2015) Fauna Europaea: Neuropterida (Raphidioptera, Megaloptera, Neuroptera). Biodiversity Data Journal 3: e4830. 10.3897/BDJ.3.e4830 PMC4411496 25941450

[B5] Badano D Acevedo F Pantaleoni RA Monserrat VJ (2016) Myrmeleon almohadarum sp. nov., from Spain and North Africa, with description of the larva (NeuropteraMyrmeleontidae). Zootaxa 4196: 210–220. 10.11646/zootaxa.4196.2.2 27988672

[B6] Boyer F Mercier C Bonin A Taberlet P Coissac E (2014) OBITools: a Unix-inspired software package for DNA metabarcoding. Molecular Ecology Resources 16(1): 176–182. 10.1111/1755-0998.12428 25959493

[B7] Duelli P Obrist MK (2019) In search of the real *Pseudomalladaprasinus* (Neuroptera, Chrysopidae). Zootaxa 4571: 510–530. 10.11646/zootaxa.4571.4.4 31715792

[B8] Duelli P Henry CS Hayashi M Nomura M Mochizuki A (2017) Molecular phylogeny and morphology of *Pseudomallada* (Neuroptera: Chrysopidae), one of the largest genera within Chrysopidae. Zoological Journal of the Linnean Society 180: 556–569. 10.1093/zoolinnean/zlw008

[B9] Elbrecht V Leese F (2017) Validation and development of COI metabarcoding primers for freshwater macroinvertebrate bioassessment. Frontiers in Environmental Science 5: 1–11. 10.3389/fenvs.2017.00011

[B10] Engel MS Winterton SL Breitkreuz LCV (2018) Phylogeny and Evolution of Neuropterida: Where Have Wings of Lace Taken Us? Annual Review of Entomology 63(1): 531–551. 10.1146/annurev-ento-020117-043127 29324039

[B11] Ferreira S Paupério J Grosso-Silva JM Beja P (2019) DNA barcoding of *Sialis* sp. (Megaloptera) in Portugal: the missing tool to species identification. Aquatic Insects 40: 1–12. 10.1080/01650424.2019.1571612

[B12] Folmer O Black M Hoeh W Lutz R Vrijenhoek R (1994) DNA primers for amplification of mitochondrial cytochrome c oxidase subunit I from diverse metazoan invertebrates. Molecular marine biology and biotechnology 3: 294–299. 7881515

[B13] Hebert PDN Gregory TR (2005) The Promise of DNA Barcoding for Taxonomy. Systematic Biology 54: 852–859. 10.1080/10635150500354886 16243770

[B14] Hebert PDN Cywinska A Ball SL DeWaard JR (2003) Biological identifications through DNA barcodes. Proceedings of the Royal Society B: Biological Sciences 270: 313–321. 10.1098/rspb.2002.2218 PMC1691236 12614582

[B15] Hebert PDN Stoeckle MY Zemlak TS Francis CM (2004) Identification of birds through DNA barcodes. PLoS Biology 2(10): e312. 10.1371/journal.pbio.0020312 PMC518999 15455034

[B16] Henry CS Wells MLM (2015) Courtship songs of green lacewings filmed in slow motion: how a simple vibrating structure can generate complex signals (Neuroptera: Chrysopidae: Chrysoperla). Journal of Insect Behavior 28: 89–106. 10.1007/s10905-015-9484-6

[B17] Henry CS Brooks SJ Duelli P Johnson JB (2002) Discovering the true *Chrysoperlacarnea* (Insecta: Neuroptera: Chrysopidae) using song analysis, morphology, and ecology. Annals of the Entomological Society of America 95: 172–191. 10.1603/0013-8746(2002)095[0172:DTTCCI]2.0.CO;2

[B18] Henry CS Brooks SJ Duelli P Johnson JB (2003) A lacewing with the wanderlust: The European song species “Maltese”, Chrysoperlaagilis, sp.n., of the carnea group of Chrysoperla (Neuroptera: Chrysopidae). Systematic Entomology 28: 131–147. 10.1046/j.1365-3113.2003.00208.x

[B19] Henry CS Brooks SJ Duelli P Johnson JB Wells MM Mochizuki A (2012) Parallel evolution in courtship songs of North American and European green lacewings (Neuroptera: Chrysopidae). Biological Journal of the Linnean Society 105: 776–796. 10.1111/j.1095-8312.2011.01845.x

[B20] Henry CS Brooks SJ Duelli P Johnson JB Wells MM Mochizuki A (2013) Obligatory duetting behaviour in the *Chrysoperlacarnea*-group of cryptic species (Neuroptera: Chrysopidae): Its role in shaping evolutionary history. Biological Reviews 88: 787–808. 10.1111/brv.12027 23433087

[B21] Hölzel H (1973) Zur Revision von Typen europäischer *Chrysopa*-Ar ten. Revue suisse de zoologie 80: 65–82. 10.5962/bhl.part.75938

[B22] Kumar S Stecher G Li M Knyaz C Tamura K (2018) MEGA X: Molecular evolutionary genetics analysis across computing platforms. Molecular Biology and Evolution 35: 1547–1549. 10.1093/molbev/msy096 29722887 PMC5967553

[B23] Letardi A (2019) Preliminary results of NEUIT (Barcoding of Italian Neuropterida) project. 10.5281/zenodo.3569411

[B24] Letardi A Almeida JM (2013) Contributing to a checklist of Neuropterida in Portugal: the Naturdata project. Açoreana 9: 29–38.

[B25] Lourenço P Brito C Backeljau T Thierry D Ventura MA (2006) Molecular systematics of the *Chrysoperlacarnea* group (Neuroptera: Chrysopidae) in Europe. Journal of Zoological Systematics and Evolutionary Research 44: 180–184. 10.1111/j.1439-0469.2006.00352.x

[B26] Machado RJP Gillung JP Winterton SL Garzón-Orduña IJ Lemmon AR Lemmon EM Oswald JD (2019) Owlflies are derived antlions: anchored phylogenomics supports a new phylogeny and classification of Myrmeleontidae (Neuroptera). Systematic Entomology 44(2): 418–450. 10.1111/syen.12334

[B27] McLachlan R (1886) Notes concerning *Chrysopaventralis*, *prasina*, *abdominalis*, *aspersa*, and *zelleri*. The Entomologist’s Monthly Magazine 23: 33–36.

[B28] Meyer M Kircher M (2010) Illumina sequencing library preparation for highly multiplexed target capture and sequencing. Cold Spring Harbor Protocols 5. 10.1101/pdb.prot5448 20516186

[B29] Monserrat VJ (2014a) Los berótidos de la Península Ibérica (Insecta: Neuropterida: Neuroptera: Berothidae). Heteropterus Revista de Entomología 14: 31–54. 10.3989/graellsia.2014.v70.111

[B30] Monserrat VJ (2014b) Los diláridos de la Península Ibérica (Insecta: Neuropterida: Neuroptera: Dilaridae). Heteropterus Revista de Entomología 14: 187–214. 10.3989/graellsia.2014.v70.111

[B31] Monserrat VJ (2014c) Los mantíspidos de la Península Ibérica y Baleares (Insecta, Neuropterida, Neuroptera, Mantispidae). Graellsia 70: e012. 10.3989/graellsia.2014.v70.115

[B32] Monserrat VJ (2016a) Los coniopterígidos de la Península Ibérica y Islas Baleares (InsectaNeuropterida, Neuroptera: Coniopterygidae). Graellsia 72: e047. 10.3989/graellsia.2016.v72.157

[B33] Monserrat VJ (2016b) Los crisópidos de la Península Ibérica y Baleares (Insecta, Neuropterida, Neuroptera: Chrysopidae). Graellsia 72: e037. 10.3989/graellsia.2016.v72.143

[B34] Monserrat VJ Acevedo F (2012a) Los ascaláfidos de la Península Ibérica y Baleares (Insecta : Neuroptera: Ascalaphidae). Heteropterus Revista de Entomología 12: 33–58.

[B35] Monserrat VJ Acevedo F (2012b) Los nemoptéridos y crócidos de la Península Ibérica (Insecta: Neuroptera: Nemopteridae, Crocidae). Heteropterus Revista de Entomología 12: 231–255.

[B36] Monserrat VJ Acevedo F (2013) Los mirmeleóntidos (hormigas-león) de la Península Ibérica y Islas Baleares (Insecta, Neuropterida, Neuroptera, Myrmeleontidae). Graellsia 69: 283–321. 10.3989/graellsia.2013.v69.098

[B37] Monserrat VJ Papenberg D (2015) Los rafidiópteros de la Península Ibérica (Insecta, Neuropterida: Raphidioptera); The snake-flies from the Iberian Peninsula (Insecta, Neuropterida: Raphidioptera). Graellsia 71(1): e024. 10.3989/graellsia.2015.v71.116

[B38] Monserrat VJ Badano D Acevedo F (2014) Nuevos datos de ascaláfidos para la Península Ibérica, con una nueva especie para la fauna europea (Insecta: Neuropterida: Neuroptera: Ascalaphidae). Heteropterus Revista de Entomología 14: 147–167.

[B39] Monserrat VJ Triviño V (2013) Atlas of the Iberian and Balearic lacewings (Insecta, Neuroptera: Megaloptera, Raphidioptera, Planipennia) Sociedad Entomologica Aragonesa. Monografias S.E.A. 13: 1–154.

[B40] Morinière J Hendrich L Hausmann A Hebert PD Haszprunar G Gruppe A (2014) Barcoding fauna bavarica: 78% of the neuropterida fauna barcoded! PLoS ONE 9(10): e109719. 10.1371/journal.pone.0109719 PMC4186837 25286434

[B41] Oliveira D Ferreira S (2020) Wesmaelius (Kimninsia) nervosus (Fabricius, 1793) (Neuroptera, Hemerobiidae) a new species of brown lacewing for the Portuguese fauna. Boletín de la Asociación Española de Entomología 44: 1–4.

[B42] Pantaleoni RA Badano D (2012) *Myrmeleonpunicanus* n. sp., a new pit-building antlion (Neuroptera myrmeleontidae) from sicily and pantelleria. Bulletin of Insectology 65: 139–148.

[B43] Papenberg D (2015) Revisión de los rafidiópteros (insectos neuropteroides, raphidiopteros) de la Península Ibérica. Universidad Complutense de Madrid.

[B44] Paupério J Fonseca N Egeter B Galhardo M Ferreira S Oxefelt F Aresta S Martins F Mata V Veríssimo J Puppo P Pinto JC Chaves C Garcia-Raventós A Peixoto S Vasconcelos LP da SS Gil P Khalatbari L Jarman S Beja P (2018) EnvMetaGen Deliverable 4.4 (D4.4) Protocol for next-gen analysis of eDNA samples. 10.5281/zenodo.2586885

[B45] Price BW Henry CS Hall AC Mochizuki A Duelli P Brooks SJ (2015) Singing from the grave: DNA from a 180 year old type specimen confirms the identity of *Chrysoperlacarnea* (Stephens). PLoS ONE 10(4): e0121127. 10.1371/journal.pone.0121127 PMC4390323 25853856

[B46] Puillandre N Lambert A Brouillet S Achaz G (2012) ABGD, Automatic Barcode Gap Discovery for primary species delimitation. Molecular Ecology 21: 1864–1877. 10.1111/j.1365-294X.2011.05239.x 21883587

[B47] Ratnasingham S Hebert PD (2007) BOLD: The Barcode of Life Data System. Molecular Ecology Notes 7: 355–364. 10.1111/j.1471-8286.2007.01678.x 18784790 PMC1890991

[B48] Shokralla S Porter TM Gibson JF Dobosz R Janzen DH Hallwachs W Golding GB Hajibabaei M (2015) Massively parallel multiplex DNA sequencing for specimen identification using an Illumina MiSeq platform. Scientific Reports 5: e9687. 10.1038/srep09687 PMC4401116 25884109

[B49] Swofford DL (2003) PAUP*. Phylogenetic analysis using parsimony (* and other methods). Sinauer Associates.

[B50] Thierry D Cloupeau R Jarry M Canard M (1998) Discrimination of the West-Palaearctic *Chrysoperla* Steinmann species of the *carnea* Stephens group by means of claw morphology (Neuroptera, Chry sopidae), 255–262.

[B51] Thompson DJ Higgins DG Gibson TJ (1994) CLUSTAL W: improving the sensitivity of progressive position-specific gap penalties and weight matrix choice multiple sequence alignment through sequence weighting, Julie. European Molecular Biology Laborator 22: 4673–4680. 10.1093/nar/22.22.4673 PMC308517 7984417

[B52] Vasilikopoulos A Misof B Meusemann K Lieberz D Flouri T Beutel RG Niehuis O Wappler T Rust J Peters RS Donath A Podsiadlowski L Mayer C Bartel D Böhm A Liu S Kapli P Greve C Jepson JE Liu X Zhou X Aspöck H Aspöck U (2020) An integrative phylogenomic approach to elucidate the evolutionary history and divergence times of Neuropterida (Insecta: Holometabola). BMC Evolutionary Biology 20: e64. 10.1186/s12862-020-01631-6 PMC7268685 32493355

[B53] Wang Y Liu X Garzón-Orduña IJ Winterton SL Yan Y Aspöck U Aspöck H Yang D (2017) Mitochondrial phylogenomics illuminates the evolutionary history of Neuropterida. Cladistics 33(6): 617–636. 10.1111/cla.12186 34724753

[B54] Winterton SL Hardy NB Wiegmann BM (2010) On wings of lace: Phylogeny and Bayesian divergence time estimates of Neuropterida (Insecta) based on morphological and molecular data. Systematic Entomology 35: 349–378. 10.1111/j.1365-3113.2010.00521.x

[B55] Winterton SL Lemmon AR Gillung JP Garzon IJ Badano D Bakkes DK Breitkreuz LCV Engel MS Lemmon EM Liu X Machado RJP Skevington JH Oswald JD (2018) Evolution of lacewings and allied orders using anchored phylogenomics (Neuroptera, Megaloptera, Raphidioptera). Systematic Entomology 43: 330–354. 10.1111/syen.12278

[B56] Yi P Yu P Liu J Xu H Liu X (2018) A DNA barcode reference library of Neuroptera (Insecta, Neuropterida) from Beijing. ZooKeys 807: 127–147. 10.3897/zookeys.807.29430 PMC6305355 30595654

